# Assessing heat exposure and its effects on farmer health, harvest yields, and nutrition: a study protocol for Burkina Faso and Kenya

**DOI:** 10.1080/16549716.2025.2513719

**Published:** 2025-07-14

**Authors:** Sandra Barteit, Windpanga Aristide Ouédraogo, Charlotte Müller, Pascal Zabré, Issouf Traoré, Valentin Boudo, Ali Sié, Guillaume Compaoré, Lucienne Ouèrmi, Stephen Munga, David Obor, Aditi Bunker, Hanns-Christian Gunga, Kristine Belesova, Till Bärnighausen, Jonas Franke, Maximilian Schwarz, Martina Anna Maggioni, Rainer Sauerborn

**Affiliations:** a Heidelberg Institute of Global Health (HIGH), Faculty of Medicine and University Hospital, Heidelberg University, Heidelberg, Germany; b Centre de Recherche en Santé de Nouna (CRSN), Institut National de Santé Publique (INSP), Nouna, Burkina Faso; c Institut Universitaire de Formations Initiale et Continue (IUFIC), Université Thomas SANKARA, Ouagadougou, Burkina Faso; d Kenya Medical Research Institute, Center for Global Health Research, Kisumu, Kenya; e Charité - Universitätsmedizin Berlin, Institute of Physiology, Center for Space Medicine and Extreme Environments Berlin, Berlin, Germany; f Department of Public Health, Environments and Society and Centre on Climate Change and Planetary Health, London School of Hygiene and Tropical Medicine (LSHTM), London, UK; g Africa Health Research Institute (AHRI), KwaZulu-Natal, South Africa; h Harvard Center for Population and Development Studies, Cambridge, MA, USA; i Remote Sensing Solutions GmbH, Munich, Germany; j Department of Biomedical Sciences for Health, Università degli Studi di Milano, Milano, Italy

**Keywords:** Stig Wall, Climate change, agricultural productivity, heat stress, outdoor worker capacity, child nutrition, remote sensing, wearable technologies, adaptation, Burkina Faso, Sub-Saharan Africa

## Abstract

Rising temperatures in Africa present an increasing threat to agricultural productivity and public health, particularly among subsistence farming communities reliant on rain-fed agriculture. Heat exposure can impair farmers’ work capacity, disrupt harvests, and heighten health risks, especially for young children vulnerable to undernutrition. The Heat to Harvest (H2H) study investigates how environmental heat exposure influences farmers’ physiological and behavioral responses, and how these in turn affect harvest yields and child nutrition. It also examines differences in labor performance and recovery between households with and without cool roof coatings, although this intervention is not the central focus. H2H is designed as a prospective cohort study nested within two Health and Demographic Surveillance Systems (HDSS) in Nouna, Burkina Faso, and Siaya, Kenya. The study integrates environmental monitoring (temperature and humidity sensors used to compute Wet Bulb Globe Temperature), biometric data (via wearables tracking heart rate, temperature, physical activity, energy expenditure, and sleep), and GPS tracking (capturing spatial mobility and labor duration). The study is embedded within a larger cluster-randomized controlled trial, facilitating comparative analysis under varying thermal conditions. Findings will provide evidence-based insights into how climate-related heat stress affects health and agricultural outcomes, supporting the development of targeted adaptation strategies to enhance resilience, health, and food security in vulnerable farming communities.

## Paper context

**Main focus:**

This methods paper outlines the approach to studying heat exposure’s impact on subsistence farmers’ work capacity in Burkina Faso and Kenya, and its effects on crop yields and child nutrition.

**Added knowledge:**

It introduces a novel framework combining wearable technology and remote sensing to assess the physiological and agricultural effects of heat stress in vulnerable farming communities.

**Global health impact for policy and action:**

The study aims to inform climate-resilient agricultural policies and heat-adaptation strategies to protect farmer health and ensure food security in regions facing increasing heat exposure.

## Background

Burkina Faso and Kenya are highly vulnerable to climate change due to their reliance on rain-fed agriculture, which supports a significant portion of their populations and serves as a fundamental pillar of their economies [1–4]. Both countries exhibit low adaptive capacity, driven by limited economic resources for implementing adaptation strategies, a high prevalence of climate-sensitive diseases, and rapid population growth, which exacerbates competition for already scarce resources such as arable land and water [5–10]. The agricultural sector, a cornerstone of economic stability in both countries, faces considerable challenges due to its lack of diversification, making it highly susceptible to climate variability [11,12]. Subsistence farming is the predominant agricultural model, with approximately 86% of households relying on their own agricultural yields for food security [13,14]. This dependence renders farming communities particularly vulnerable to erratic weather patterns and limits their financial capacity to buffer against poor harvests. Climate change further exacerbates agricultural output declines, food insecurity, and nutritional deficiencies [15]. In Burkina Faso, land degradation is a pressing issue, with approximately 33% of land classified as degraded. The country loses an estimated 360,000 hectares to desertification annually, equivalent to 1.3% of its total land area [16]. Additionally, climate change is accelerating soil nutrient depletion and altering plant physiology in both Burkina Faso and Kenya, leading to reduced crop nutrient content and worsening malnutrition [17]. Climate change also drives an increase in pest populations and the spread of plant diseases, further compromising crop yields, while heightened evaporation rates reduce water availability for agriculture [18–20]. Projections for West Africa indicate that climate change could lead to substantial declines in crop yields by 2050, with anthropogenic influences already contributing to shifts in microclimatic conditions [21–25]. Both Burkina Faso and Kenya have experienced rising temperatures, with Burkina Faso recording an average temperature increase of approximately 1°C since the early 2000s [26]. This warming trend has been accompanied by more frequent and intense heatwaves and extreme rainfall events, contributing to significant reductions in regional crop yields. Simulation models predict millet yield losses of 10–20% and sorghum losses of 5–15% under current climate change trajectories [26].

The health impacts of climate change are primarily driven by rising temperatures and increased heat exposure, which are projected to contribute to a surge in heat-related morbidity and mortality, including conditions such as heat stress, cardiovascular diseases, and renal dysfunction [9]. Additionally, climate change is expected to exacerbate the burden of communicable diseases such as dengue fever, pathogenic *Vibrio* infections, and malaria, as warmer and more humid conditions enhance the suitability for vector proliferation and disease transmission [9]. Changes in rainfall and temperature patterns are likely to affect mosquito breeding cycles, further increasing malaria risk [9]. Notably, malaria, pneumonia, and diarrheal diseases together account for nearly half of all deaths in children under five in sub-Saharan Africa (SSA) [27]. In the Kossi region of northwestern Burkina Faso, shifting seasonal weather patterns, rising temperatures, and extreme weather events, including heavy rainfall and flooding, have been associated with direct health impacts, including increased incidences of heatstroke and malaria, as well as indirect effects such as heightened food insecurity and child undernutrition [3,28–32]. In both Burkina Faso and Kenya, climate change exacerbates child undernutrition by compromising food security [30,33,34]. The increasing frequency of severe weather events, such as droughts and floods, reduces crop yields and elevates food prices, leading to inadequate dietary intake and heightened health risks associated with undernutrition [30,33,34]. Chronic undernutrition affects 26% of children under five in Burkina Faso and over 25% in Kenya, contributing to stunting, while acute undernutrition leads to wasting in about 7% of children in Burkina Faso and 4% in Kenya. These conditions have long-term consequences for cognitive and physical development [30]. Moreover, exposure to drought during late childhood and early adolescence has been shown to have persistent adverse effects on growth, primarily mediated by reduced nutritional intake during critical developmental years [35]. In Burkina Faso, studies have demonstrated that rainfall variability directly impacts crop yields and correlates with increased child undernutrition in affected households [36,37].

Despite growing recognition of the impacts of climate change, empirical research on the pathways linking human heat exposure to agricultural outcomes and individual physiological responses remains limited. Comprehensive studies investigating the effects of heat stress on harvest yields, soil quality, plant physiology, livestock health, and farmers’ work capacity are lacking, particularly in SSA [22,23,38,39]. The cascading impacts of heat-induced physiological strain on labor capacity, agricultural productivity, and downstream consequences such as child nutrition are poorly characterized. Moreover, the degree to which these effects are already manifesting in vulnerable populations, and the extent of intra-population variability, remain insufficiently studied. This knowledge gap is largely attributable to limited data availability, underscoring the need for context-specific, empirically grounded research to inform effective adaptation strategies in SSA [40].

To address these gaps on the effects of heat exposure on agricultural productivity and health, the Heat to Harvest (H2H) study investigates the following research questions and corresponding hypotheses.

1. **What is the impact of heat exposure on the physiological responses of farmers?**
  - **Hypothesis 1**: Increased heat exposure will significantly elevate physiological strain, as measured by heart rate and body temperature.
2. **How does heat exposure influence farmers’ work capacity and farming behaviors?**
  - **Hypothesis 2**: Higher temperatures will reduce work capacity and alter farming behaviors, including daily activity patterns, sleep quality, duration, and energy expenditure.
3. **What is the relationship between heat exposure, work capacity, health, and farming practices on harvest yields?**
  - **Hypothesis 3**: Heat exposure will lead to reduced harvest yields by impairing farmers’ work capacity and health.
4. **How do these factors indirectly affect the nutritional status of children under five in farming households?**
  - **Hypothesis 4**: Reduced work capacity and lower harvest yields due to heat exposure will negatively impact child nutrition.

This study systematically investigates these pathways using wearable sensors, GPS tracking, offering valuable insights into climate-related challenges faced by subsistence farmers and their families.

## Methods

### Health impact pathways of heat on physiological response, agricultural capacity, harvest yield, and nutritional status of children

The first health impact pathway examines physiological strain due to heat exposure, incorporating temperature, humidity, and the Wet Bulb Globe Temperature (WBGT) index. The WBGT is a standardized composite index that quantifies environmental heat stress by integrating multiple meteorological parameters, including air temperature, relative humidity, and solar radiation. It serves as a physiologically relevant proxy for the subjective perception of heat, capturing the cumulative thermal load experienced by the human body under varying environmental conditions, and is widely employed to assess the potential impact of heat on physical performance and safety. As WBGT levels increase, physical work capacity declines, with significant reductions observed at 27–28°C WBGT [38,41,42].

The second health impact pathway examines the relationship between heat exposure, physiological responses, and work capacity. Prolonged exposure to heat increases cardiovascular strain (above ~26–27°C cardiovascular strain can begin to increase), exacerbates pre-existing health conditions, and heightens the risk of heat-related illnesses, including heat stress, heat stroke, and renal failure. These health effects contribute to reduced work performance, affecting the duration and intensity of agricultural labor. Given that heart rate fluctuations result from both physical exertion and heat stress, careful methodological adjustments will be made to differentiate between these factors (e.g., accounting for cardiac drift in wearable device data).

The third health impact pathway examines how heat affects agricultural work capacity, particularly during peak farming periods such as harvesting. Heat exposure may lead to shortened work shifts, more frequent rest breaks, and reduced overall labor productivity, ultimately impacting both crop yields and harvest quality. This pathway investigates the interplay between heat stress, labor dynamics, and agricultural output, while accounting for variations in socioeconomic conditions, farming techniques, and land use.

The fourth health impact pathway addresses the indirect consequences of heat-induced agricultural disruptions on child nutrition. Declining crop yields and increasing food insecurity may lead to dietary deficiencies, heightened undernutrition, and associated health risks in children under five. By linking environmental stressors to household food availability, this pathway seeks to quantify the nutritional and health impacts of heat exposure on vulnerable populations.

**Figure 1.**
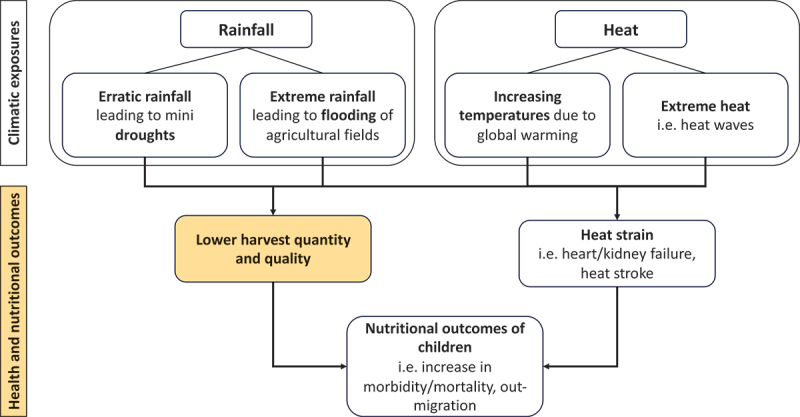
Conceptual framework of the H2H study.

**Figure 2.**
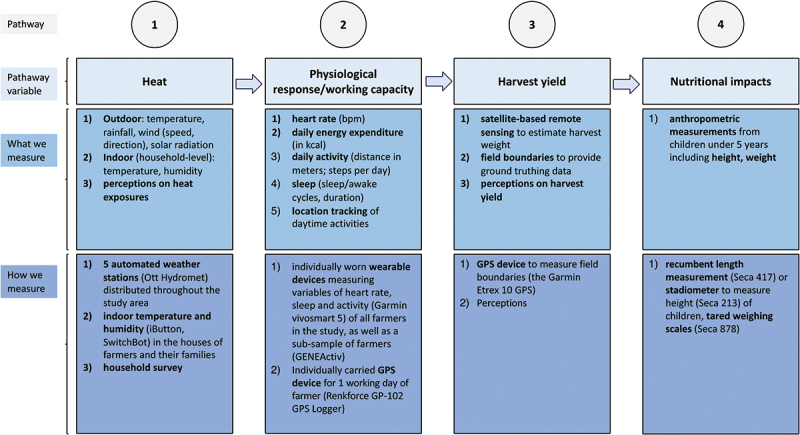
Pathway of the H2H study, following four health impact pathways: in this study we focus mainly on heat exposure, but also consider the variable of rainfall, its impact on work capacity, physiological responses in farmers, outcomes on harvest yield, and nutritional status in children under age 5.

A conceptual framework (refer to Figures 1 and 2 for the conceptual framework of the H2H study and its pathways) illustrates the relationships between heat exposure, physiological strain, agricultural productivity, and child nutrition. The study will incorporate controls for potential confounders to ensure that observed effects can be robustly attributed to heat exposure rather than external factors such as soil fertility, rainfall variability, or market fluctuations.

### Study design

**Figure 3.**
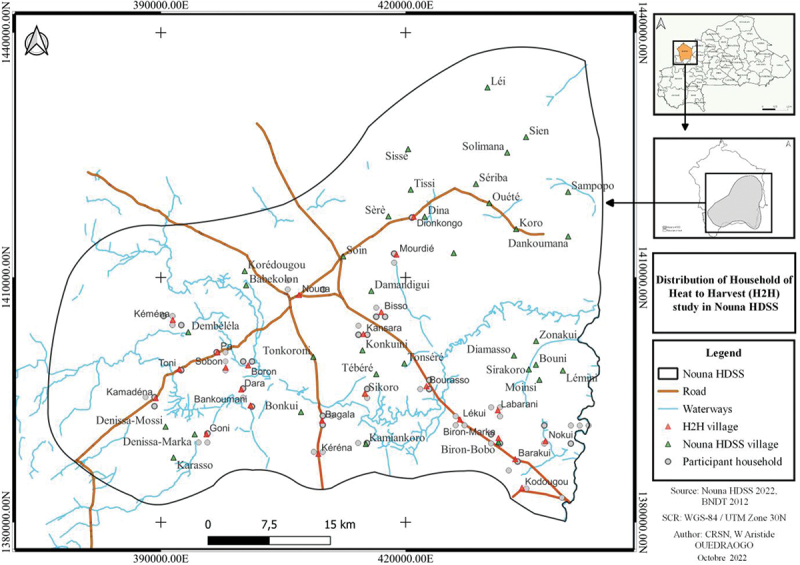
Study area (health demographic Surveillance System (HDSS)) with the H2H village in the Kossi province. For the study area of the Siaya HDSS, refer to [43]. Map was generated using QGIS (version 3.16.14; QGIS development team, 2021). QGIS is an open-source geographic information System that supports viewing, editing, and analysis of geospatial data. The software can be accessed and downloaded at https://qgis.org.

The H2H study will use a prospective cohort design implemented within two established Health and Demographic Surveillance Systems (HDSS): the Nouna HDSS in Burkina Faso (see Figure 3) and the Siaya HDSS in Kenya (see [43] for further details). The Siaya HDSS, operational since 1990, covers a population of about 220,000 across 385 villages in Siaya County, Kenya, an area with a tropical climate, seasonal rainfall (including heavy ‘long’ rains from March to May and ‘short’ rains between October and December), and significant health challenges including endemic malaria, and high rates of HIV and tuberculosis [43,44]. The Nouna HDSS, in operation since 1992, longitudinally observes health and demographic changes among 124,957 individuals in 59 villages and the city of Nouna, Burkina Faso [44]. This area, 300 km northwest of Ouagadougou, experiences a Sudano-Sahelian climate with a long dry season and a short rainy season, receiving an average annual rainfall of 700 mm, with over 30% in August alone [44,45].

### Study participants and sample size

Participants will be randomly selected from both HDSS sites and will include subsistence farmers as well as children residing in their households. A farmer is defined as an individual whose primary occupation is agriculture, evidenced by the active cultivation of at least one food crop, with household livelihood primarily dependent on agricultural production.

The primary outcome variable is heart rate (beats per minute, bpm), used as a physiological proxy for heat-related strain in farmers. Heart rate reflects both physical exertion during agricultural labor and cardiovascular responses to heat stress, making it a key metric for assessing workload intensity and thermal burden. However, as heart rate increases can result from both physical exertion and heat exposure, the study will account for cardiac drift, which refers to the gradual increase in heart rate over time due to prolonged exertion in hot conditions. To differentiate between workload-related heart rate elevations and heat-induced cardiovascular strain, the following strategies will be applied:

- Step data and GPS tracking will provide context for exertion levels during work activities.
- Time-of-day analysis will help distinguish between work-related and heat-related heart rate increases.
- Cross-referencing heart rate with environmental data (WBGT, temperature, humidity) will enable more precise differentiation between exertion-driven and heat-induced cardiac responses.

To estimate an appropriate sample size for the H2H pilot study, a power analysis is conducted for comparing means between two independent groups:

$$n=\frac{2{\left(Z_{\alpha /2}+Z_{\beta }\right)}^{2}\times \sigma^{2}}{\delta^{2}}$$

$$n=\frac{2{\left(Z_{\alpha /2}+Z_{\beta }\right)}^{2}\times \sigma^{2}}{\delta^{2}}$$

Where:

- n is the sample size required per group.
- Z_α/2_ is the critical value associated with a desired alpha level (commonly set at 0.05 for a 95- Z_β_ is the critical value associated with the desired power of the test (commonly set at 0.80, yielding a Z_β_ value of 0.84).
- σ is the standard deviation of the outcome variable, which in this case is the bpm difference.
- δ is the expected difference in means between the two groups.

A sample size of 63 participants per group is estimated to detect a mean difference of 5 bpm in heart rate with 95% confidence and 80% statistical power. To accommodate potential attrition and inter-individual variability, the H2H study will enroll a total of 210 participants across 25 villages within the HDSS sites during the feasibility phase.

In the Nouna HDSS, the H2H study is nested within a cluster randomized controlled trial (cRCT) evaluating the effectiveness of cool roof coatings as a passive home cooling intervention (DRKS trials registry number DRKS00023207) [46]. Cool roof coatings are designed to reflect solar radiation and exhibit high thermal emittance, thereby reducing indoor temperatures [47].

The cRCT includes 600 households, with 300 households randomly assigned to receive the cool roof intervention and 300 serving as controls. For the H2H study, farmers will be equally sampled from both intervention and control households to ensure balanced exposure conditions. The H2H study does not treat the cool roof intervention as a direct proxy for heat exposure but considers it a potential modifier of recovery following occupational heat stress.

This nested design facilitates the investigation of both direct and indirect heat-related pathways [1]: direct exposure during agricultural work – analyzing physiological responses, work capacity, and productivity; and [2] post-exposure recovery at home, examining whether improved indoor thermal environments enhance recuperation and influence subsequent health and labour outcomes.

While the cool roof cRCT focuses on the effectiveness of home cooling, the H2H study is primarily concerned with occupational heat exposure. Nevertheless, the interaction between workplace heat stress and home-based recovery conditions is a central component in understanding the cumulative effects of heat on health and productivity over time.

### Study procedures and measurements

Participants will be monitored over a 12-week period each year during the harvest season, which coincides with the rainy season in both the Nouna and Siaya HDSS sites, for a duration of three consecutive years. This repeated seasonal monitoring enables the collection of longitudinal data on heat exposure, work capacity, and agricultural productivity, capturing both seasonal dynamics and interannual variability.

At baseline, field workers will collect socio-demographic information using structured questionnaires implemented via Survey Solutions (https://mysurvey.solutions/en/). The digital platform includes multi-level plausibility checks to enhance data quality. Data will be collected using tablets and uploaded directly to the Survey Solutions server either from the field or HDSS offices. Supervisors will review submissions for completeness and consistency; if discrepancies or missing values are identified, forms will be returned to field workers for verification and correction prior to final approval.

Before the onset of participant monitoring, field workers will measure the perimeter of each farmer’s plot and record the types of crops cultivated. These data will be integrated into remote sensing models to support the estimation of harvest yields and agricultural productivity [31].

Participants aged 18 years and older will be equipped with wearable devices (see Chapter 2.4.1 onwards for further details) that continuously monitor heart rate, physical activity, and sleep patterns throughout the study period. These devices will generate high-resolution data on physiological strain, behavioral activity, and recovery dynamics in response to heat exposure, enabling detailed analysis of the relationship between thermal stress and work capacity. Participants are instructed to wear the devices continuously, with data synchronized via the Fitrockr application (https://www.fitrockr.com/). Due to limited smartphone access among participants, field workers will oversee data synchronization using tablets to ensure regular uploads and minimize data loss.

Complementing the wearable monitoring, each participant will carry a GPS tracker for a minimum of 20 days annually during the harvest season. The GPS devices will capture spatial and temporal work patterns, including fieldwork duration and distance traveled, offering granular insights into labor exposure under varying thermal conditions. Data from GPS units will be stored locally and synchronized offline by field staff at predefined intervals.

To ensure uninterrupted device operation, participants will be provided with power banks for overnight charging. Additionally, foldable solar panels will be supplied to allow direct charging of devices or recharging of power banks as needed.

#### Heat (health impact pathway 1)

##### Indoor air temperature and humidity

Indoor air temperature and relative humidity will be recorded at 15-minute intervals using iButton DS1923 hygrochron and SwitchBot Thermometer & Hygrometer sensors. Additionally, to validate these readings, a portable Wet Bulb Globe Temperature (WBGT) device (PCE-WB20SD, Germany) will be deployed in a subset of households. WBGT is primarily used for outdoor environmental heat stress assessments at workplaces; however, in this study, it will serve as a complementary tool to verify indoor heat conditions.

##### Outdoor weather metrics

**Figure 4.**
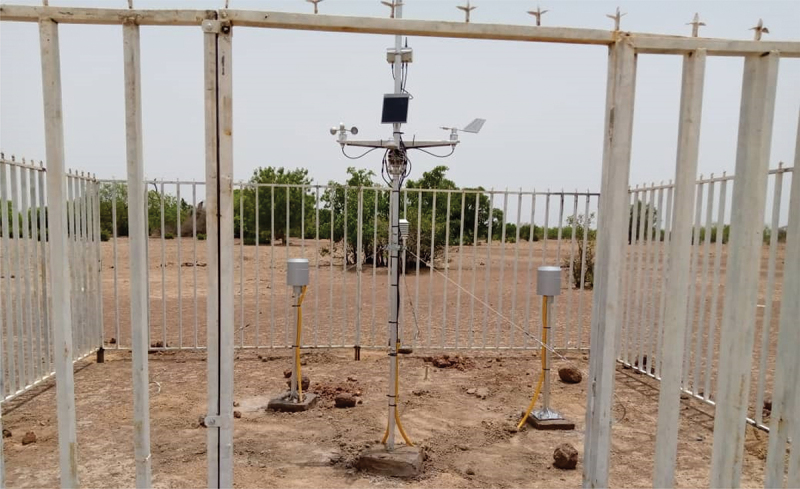
Automated weather station with General Packet Radio Service (GPRS) connectivity located within the Nouna Health and Demographic Surveillance System (HDSS) site, recording real-time data on rainfall, temperature, wind speed and direction, and solar radiation.

The Nouna and Siaya HDSS areas each have five automatic weather stations that have been operational since mid-2020 (as depicted in Figure 4). These stations continuously record meteorological parameters, including:

- Air temperature
- Rainfall
- Wind speed and direction
- Solar radiation

These data points are updated every 15 minutes and transmitted in real time via a General Packet Radio Service (GPRS) system, ensuring immediate remote access for analysis. The recorded data is stored on a dedicated server, which also provides a dashboard for visualization (for specific locations of weather stations, see reference [45].

#### Physiological response and work capacity (health impact pathway 2)

To monitor farmers’ physiological responses to heat exposure, the study will deploy two types of wearable devices to enable continuous, high-resolution data collection. All participants will wear the Garmin Vivosmart 5, which records heart rate, step count, and sleep metrics, including duration and quality. This device will be worn throughout the study period to provide real-time data on physiological strain. Additionally, a subset of participants will wear the Activinsights GENEActiv Original, a high-resolution actigraphy device that records gross motor activity and sleep at 10 hz, providing complementary data to the Vivosmart 5. To distinguish between heart rate changes due to physical exertion and those due to heat exposure, the analysis will incorporate methodological corrections, including adjustments for cardiac drift.

To assess work capacity, daily activity, and rest behavior, the study will monitor key indicators such as time engaged in fieldwork, distance traveled, and frequency and duration of rest periods. Each participant will use a Renkforce GP-102 Porter GPS tracker for a minimum of 20 days during the harvest season, capturing spatial and temporal data on labor patterns. GPS data will be stored locally and synchronized offline by field workers at scheduled intervals.

Prior to deployment, compatibility testing will be conducted to ensure that concurrent use of multiple wearable devices does not result in signal interference. Charging logistics will be optimized by providing participants with power banks and foldable solar panels, enabling overnight charging and minimizing disruptions to continuous data collection.

#### Harvest yield (health impact pathway 3)

Remote sensing data will be used to estimate harvest yields, based on a previously developed and ground-truth validated model [31,48] in the Nouna HDSS. The remote-sensing based model will be adapted to the Kenyan context as a part of the H2H study. Specifically, Sentinel-2 satellite imagery will be processed for the growing seasons of five key crops. To monitor vegetation health and estimate household-level yields, three monthly vegetation indices will be calculated: the Normalized Difference Vegetation Index (NDVI), the Normalized Difference Red Edge Index (NDRE), and the Normalized Difference Water Index (NDWI). These indices, combined with rainfall data, will serve as input parameters for LASSO (Least Absolute Shrinkage and Selection Operator) regression models. A distinct model will be developed for each crop to generate crop-specific yield estimates. For further details, see [31].

Field boundaries will be mapped using GPS devices (Garmin Etrex 10, Garmin USA), with measurements conducted on two separate occasions per field. Field workers will accompany farmers around each field’s perimeter to ensure accurate boundary delineation. This method will enable precise estimation of household-level agricultural output by crop type, while also geolocating fields for linkage to household data.

#### Anthropometric measurements (health impact pathway 4)

Anthropometric measurements will be collected from children under five years of age to assess the prevalence of undernutrition in relation to household agricultural production. Interviewers will use tared weighing scales, stadiometers, and length boards (all provided by Seca, Germany) to measure weight, standing height, and recumbent length, respectively. Each measurement will be taken twice by trained interviewers, with discrepancies checked and the mean value used for analysis. Detailed protocols follow those of Mank et al., who conducted anthropometric assessments in the same study region between 2017 and 2019 [30,49]. All measurements will be taken at the beginning of the harvest season, a period typically marked by reduced household food availability and increased food insecurity.

All measurements will be taken at the onset of the harvest season, a time typically characterized by diminished household food availability and heightened food insecurity.

### Data analysis

To examine the cascading effects of heat exposure on farmers’ work capacity and its subsequent impact on child nutrition, we will apply a multi-level analytical approach. Descriptive statistics – including measures of central tendency (mean, median) and dispersion (standard deviation, interquartile range) – will be calculated for each variable. Data distribution, outliers, and potential correlations will be visually assessed using histograms, box plots, and scatter plots.

**Figure 5.**
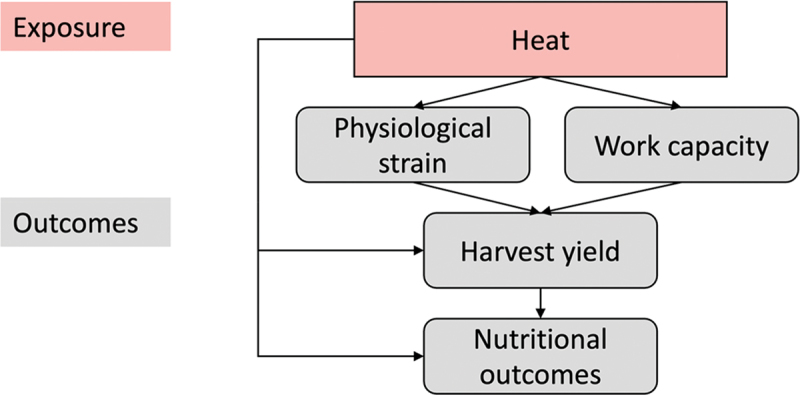
Overview of exposure and outcome variables within the Heat to Harvest (H2H) study, and their sequential and cascading framework.

Analyses will be stratified by age and gender to explore subgroup-specific differences in the effects of heat exposure. The analytical framework – detailing variables, data sources, measurement units, and their classification as continuous or categorical – is presented in Table 1. In addition, a Directed Acyclic Graph (DAG) will be constructed to visualize causal relationships and confounding factors affecting work capacity, agricultural productivity, and child nutritional status (see analytical framework: Figure 5 and Table 1).

**Table 1.** Overview of pathways, outcomes and exposure with corresponding measured variables.

	**Pathway**	**Measured variable**	**Functional form**
Exposure	Heat	– Indoor temperature and humidity	– continuous
		– Outdoor climate conditions (air temperature, rainfall, wind speed, wind direction, solar radiation, humidity)	– continuous
Outcome	Physiological response and work capacity	– Wearable data (heart rate, energy expenditure, activity levels, sleep)	– continuous
		– GPS data (farmers work itineraries and time allocation)	– spatiotemporal data
Outcome	Harvest yield	– Remote sensing data (vegetation indices)	– raster
		– Field boundaries measured with GPS devices (locations of crop fields)	– point data
Outcome	Nutritional status	– Anthropometric measurements (weight, height measurements of children < 5 years)	– continuous

#### Structural equation modeling (SEM) and mediation analysis

To assess both direct and indirect effects of heat exposure, the study will employ Structural Equation Modeling (SEM) with mediation analysis. This approach enables the simultaneous estimation of multiple pathways linking heat exposure to physiological stress, work capacity, harvest yields, and child nutrition, providing a comprehensive understanding of the cascading impacts of heat stress.

Direct effects will be assessed by evaluating the independent influence of heat exposure on physiological stress (e.g. heart rate, energy expenditure), work capacity (e.g. labor duration, frequency of rest breaks), and child nutritional status. These analyses will offer insights into the immediate physiological and occupational impacts of heat and their direct links to health outcomes in children.

Beyond direct effects, SEM will estimate indirect pathways through which heat exposure impacts agricultural productivity and child nutrition. The central hypothesis posits that heat exposure induces physiological stress, which subsequently reduces work capacity, leading to decreased harvest yields and, ultimately, to poorer nutritional outcomes in children. Mediation will be tested using specific pathway models, such as:

- Heat exposure → Physiological stress → Reduced work capacity
- Heat exposure → Reduced work capacity → Lower harvest yields → Poorer child nutrition

To quantify the role of mediating variables, indirect effects (e.g. via work capacity) will be compared with total effects (the overall impact of heat exposure on child nutrition), allowing for estimation of the relative contribution of different pathways.

To ensure the validity and robustness of the SEM, model fit will be assessed using multiple indices, including the Chi-square test, Comparative Fit Index (CFI), Tucker-Lewis Index (TLI), and Root Mean Square Error of Approximation (RMSEA). Through this rigorous analytical framework, the study aims to generate reliable and generalizable evidence on how heat stress affects the livelihoods and health of subsistence farming households.

#### Advanced analytical methods

Given the complex and non-linear interactions between heat exposure, agricultural productivity, and health outcomes, the study will complement traditional statistical approaches with selected applications of artificial intelligence (AI).

- Artificial Neural Networks (ANNs) will be applied to integrated datasets that include indoor temperature, heart rate, anthropometric data, and crop yields to model physiological stress and child nutritional status. These models are well-suited to detect non-linear relationships and interactions among diverse inputs and may offer predictive insights that complement regression-based inference.
- Recurrent Neural Networks (RNNs), particularly Long Short-Term Memory (LSTM) networks, will be used to analyze temporal dynamics in time-series data, including heart rate, ambient and indoor temperatures, and work/rest cycles. These models will help identify lagged effects of heat stress, such as cumulative physiological burden or delayed impacts on productivity and nutritional outcomes.

To ensure robustness and interpretability, all models will be evaluated using cross-validation techniques and compared against traditional statistical benchmarks. Where applicable, we will explore the use of interpretable AI methods (e.g., SHAP) to identify key drivers and support policy-relevant insights.

#### Heat – outdoor and indoor temperatures and humidity (health impact pathway 1)

To evaluate how housing characteristics and microclimatic conditions influence individual heat exposure, we will conduct a descriptive analysis of indoor and outdoor temperature and humidity, with a particular focus on differences between households with and without cool roofs. This analysis will quantify thermal and humidity gradients across housing types and environments, offering insight into how structural factors influence indoor microclimates and outdoor heat stress conditions.

To ensure a comprehensive characterization of environmental exposure, the following components will be examined:

- Daily and seasonal temperature fluctuations, enabling the identification of peak heat stress periods across the agricultural calendar.
- Indoor – outdoor temperature and humidity differentials, stratified by roof type, ventilation characteristics, and other household attributes, to assess how built environments mediate exposure.
- The influence of humidity, which plays a critical role in perceived heat stress and modulates physiological responses, especially under high ambient temperatures.

These environmental variables will be statistically summarized using measures of central tendency and dispersion (mean, median, standard deviation, interquartile range), and visualized via time series plots, box plots, and heat maps. Stratified comparisons will also be conducted by region, household type, and season to identify spatial and temporal patterns in heat exposure.

Findings from this microclimate analysis will be integrated into the broader analytical framework (see Figure 5 and Table 1), enabling linkage of environmental conditions with outcomes such as physiological strain, labor capacity, and agricultural productivity. This component is essential for informing both the SEM-based mediation models (Section 2.5.1) and the machine learning approaches (Section 2.5.2), providing environmental context for predictive modeling.

In addition, we will assess how natural variability in environmental parameters – including diurnal temperature ranges, humidity shifts, and extreme weather events – contributes to heat stress dynamics. These factors will be included as covariates or interaction terms in regression and SEM models, ensuring that environmental heterogeneity is appropriately accounted for in downstream analyses of heat-related impacts.

#### Work capacity and physiological response (health impact pathway 2)

To further quantify the relationship between heat exposure and farmers’ work capacity, we will apply a combination of regression models and machine learning techniques. These approaches complement the broader analytical framework by enabling the detection of both linear and non-linear associations between environmental conditions and physiological responses.

Key heat exposure variables – indoor and outdoor temperature, humidity, and Wet Bulb Globe Temperature (WBGT) – will be modeled against continuous physiological indicators such as heart rate, activity levels, and sleep patterns. Linear mixed-effects models will be used to account for within-subject variability and repeated measures, with random effects specified at the individual level to capture heterogeneity in exposure and response. These models will incorporate covariates such as socioeconomic status and farming intensity to control for confounding.

Model validation will involve standard diagnostic tools including residual and Q – Q plots to assess normality, and Variance Inflation Factor (VIF) analysis to detect multicollinearity. In parallel, cross-validation and residual error analysis will be employed to test model robustness and predictive accuracy. Sensitivity analyses will explore how natural variations in environmental conditions – particularly fluctuations in temperature and humidity – affect the stability and reliability of the modeled outcomes.

To enhance the physiological assessment of energy expenditure and physical effort, we will deploy the Renkforce GP-102 GPS Logger, which includes an integrated pedometer function. These devices will collect location and movement data every 15 minutes, allowing for the estimation of energy expenditure based on distance traveled, travel speed, terrain changes, and rest intervals.

Time spent in agricultural fields will be georeferenced, with GPS data linked to each participant’s unique ID and merged with socio-demographic characteristics, field boundaries, and movement patterns. This enables a comprehensive analysis of how mobility and environmental exposure interact to shape work capacity.

Additionally, we will analyze morning travel distances between participant households and agricultural plots – both Euclidean (straight-line) and actual walking paths – to estimate variation in daily physical exertion. These spatial metrics will be evaluated in relation to ambient and nighttime temperatures, providing further insights into how environmental stressors influence the following day’s physiological readiness and labor performance.

#### Harvest yields (health impact pathway 3)

To assess spatial variation in agricultural productivity and its relationship with heat exposure, we will conduct geospatial analyses using Geographic Information System (GIS) tools. Field boundaries – mapped via GPS – will be overlaid with remote sensing data to extract vegetation indices for each field, enabling a spatially explicit assessment of crop health and yield under varying environmental conditions.

Household-level agricultural production will be estimated using LASSO (Least Absolute Shrinkage and Selection Operator) regression models, incorporating variables such as crop type, phenological stages, and time-series vegetation indices – specifically the Normalized Difference Vegetation Index (NDVI), Normalized Difference Red Edge Index (NDRE), and Normalized Difference Water Index (NDWI) – derived from Sentinel-2 satellite imagery, as well as rainfall data from CHIRPS. To improve model robustness and prevent overfitting, five-fold cross-validation will be used to optimize model parameters and assess predictive performance.

Separate LASSO models will be developed for each major crop type, following established methods [31], thereby enhancing model specificity and improving yield forecasts at the field level. This modeling approach allows us to link remote sensing indicators to field-level agricultural output with high spatial and temporal resolution.

To evaluate the influence of heat exposure on productivity, we will identify and compare high- and low-yield regions in relation to spatial and temporal variations in temperature, integrating both indoor and outdoor temperature data. Additionally, fluctuations in temperature, humidity, and extreme weather events will be included in the analysis to assess their contributions to yield variability and to control for potential environmental confounders.

By embedding these geospatial analyses within the broader analytical framework, we aim to generate nuanced insights into how environmental stressors – particularly heat – affect agricultural output at the household level, and how this, in turn, may mediate impacts on work capacity and child nutrition.

#### Nutritional status (health impact pathway 4)

Anthropometric measurements, including weight and height assessments of children under five years of age, will be used to identify those with poor nutritional status. Nutritional classification will follow the World Health Organization (WHO) Child Growth Standards, where chronic undernutrition (stunting) is defined as a height-for-age z-score (HAZ) ≤ −2.0, and acute undernutrition (wasting) as a weight-for-height z-score (WHZ) ≤ −2.0, adjusted for age and sex [50].

To assess the relationship between heat exposure and child nutritional status, chi-square tests will first be used to evaluate unadjusted associations associations between categorical heat exposure levels and the prevalence of stunting and wasting. To estimate the independent effect of heat exposure on child growth outcomes, multivariate logistic regression models will be employed, adjusting for potential confounders such as household socioeconomic status, dietary intake, and recent illness episodes. To ensure the robustness of these findings, sensitivity analyses will be conducted to control for key confounding factors, including household socioeconomic status, dietary intake, and recent illness episodes. These additional analyses will allow for a more accurate estimation of the independent effect of heat exposure on child growth outcomes, strengthening causal inference within the broader analytical framework.

### Ethics approval and consent to participate

Ethical approval for this study was granted by the Ethics Committee of the Medical Faculty at Heidelberg University in August 2021 (S-293/2019) and by the Ethics Committee of the CRSN in July 2021 (2021–010/MS/SG/INSP/CIE). We will approach all prospective participants to secure informed consent for their involvement in the study procedures. This will include their agreement to wear a GPS device during work activities and to allow GPS tracking of their agricultural fields on a single occasion. All procedures involving human data in this study will be conducted in compliance with the Declaration of Helsinki (as revised in 2013), and all methods will adhere to the relevant guidelines and regulations.

## Expected results

The H2H study aims to collect and integrate weather-related, physiological, agricultural, and nutritional data to assess the impacts of heat stress on farmers’ physiological responses, agricultural productivity, and child nutrition. By triangulating these data streams, the study seeks to identify key determinants of heat-induced reductions in work capacity, declines in crop yields, and nutritional vulnerabilities, while systematically accounting for potential confounding factors such as socioeconomic status, farm size, and household food access.

The expected outcomes include evidence-based insights to inform climate-resilient farming practices, strategies to mitigate productivity losses under heat stress, and recommendations to stabilize yields amid increasing climatic variability. Findings will support efforts to enhance farmer resilience, improve household food security, and foster sustainable agricultural adaptation.

To ensure practical applicability, the study will assess the feasibility of predictive modeling tools for early warning systems, engage with local stakeholders to facilitate knowledge transfer and implementation, and emphasize continuous monitoring and iterative refinement of adaptive practices. However, the effectiveness of these interventions will depend on both the willingness and capacity of farming communities to adopt new strategies, and the presence of institutional and policy support to enable meaningful and sustained transformation.

### Work capacity and physiological response

With rising global temperatures, we anticipate a measurable decline in the physical work capacity of study participants, particularly during periods of elevated thermal strain. This reduction is expected to manifest as shortened time spent in agricultural fields for planting and harvesting, as well as adjustments in the duration and intensity of farm labor in both Burkina Faso and Kenya. In response, farmers may adopt adaptive pacing strategies, such as modifying work schedules, shifting tasks to cooler hours, or increasing the frequency of rest breaks to mitigate heat-related stress.

Prolonged exposure to high temperatures may also elevate the risk of heat-related illnesses, including heat exhaustion, dehydration, and thermoregulatory impairments. To monitor these responses, the study will employ consumer-grade wearable devices to capture high-resolution physiological and behavioral data, including heart rate, activity levels, estimated energy expenditure, sleep duration, and sleep quality. These metrics will provide critical insights into how heat stress affects health, labor patterns, and potential individual-level coping mechanisms.

In parallel, GPS loggers will record movement trajectories and time use, enabling spatial and temporal analysis of daily routines and fieldwork modifications in response to heat exposure. Farmers are likely to face difficulties performing physically demanding tasks during peak heat hours, which may lead to reduced labor output and greater health vulnerability. By linking GPS and wearable data, we will track physiological adjustments (e.g. changes in heart rate variability or physical activity patterns) and quantify how heat exposure reshapes daily behaviors and well-being.

Recognizing the limitations of wearable devices in directly measuring outcomes such as dehydration or mental health status, the study will incorporate complementary contextual data – including self-reported symptoms, behavioral observations, and environmental parameters – to refine the interpretation of physiological signals and strengthen the assessment of heat-related stress and adaptation strategies.

### Harvest yields

By integrating remote sensing techniques with a household-level crop yield model, we will generate detailed estimates of agricultural productivity across study sites. This approach allows for a fine-grained assessment of how climatic variables – including temperature, humidity, and extreme weather events – affect crop yields over time and space.

To ensure robust and context-specific yield estimations, the model will incorporate digitized field boundary data, time-series vegetation indices (e.g. NDVI, NDRE, NDWI), and rainfall patterns. Furthermore, we will evaluate the role of non-climatic factors such as soil quality, farm management practices, and seed variety selection in contributing to observed yield differences.

The analysis will offer a comprehensive understanding of the ecological and socio-economic impacts of climate change on smallholder farming systems. These insights will be critical for identifying vulnerability hotspots, developing targeted adaptation strategies, and informing evidence-based policy interventions to strengthen climate resilience among farming communities.

### Nutritional status

To assess the nutritional impacts of heat stress on farming households, the study will analyze key anthropometric indicators, including weight and height measurements, and examine the relationship between agricultural productivity and child nutritional status across the study regions.

Effect sizes will be derived in the form of exposure – response functions (ERFs), quantifying the links between heat exposure, physiological strain, work capacity, harvest outcomes, and child nutrition. These ERFs can inform climate impact models, such as the Inter-Sectoral Impact Model Intercomparison Project (ISIMIP), enabling projections of how rising temperatures may affect health, labor productivity, and household food security under different climate scenarios.

Building on prior research that modeled the impact of climate change on child mortality under a 1.5°C global warming scenario [26], the study will generate critical insights for adaptation planning. By elucidating the pathways through which heat exposure undermines both farming productivity and nutritional outcomes, the findings will support evidence-based decision-making to inform targeted mitigation and adaptation strategies. Ultimately, this work aims to strengthen the resilience of at-risk communities facing climate-induced food insecurity.

## Discussion

The H2H study aims to deliver a comprehensive assessment of the impacts of climate change on agricultural communities in Sub-Saharan Africa (SSA). As global temperatures continue to rise, the effects on outdoor workers, particularly subsistence farmers, are expected to intensify. This study will investigate the interrelated pathways linking heat exposure, physiological responses, work capacity, agricultural productivity, and child nutrition, providing an integrated understanding of heat stress impacts on farming households.

In parallel, the study will assess whether and how farming routines adapt in response to rising temperatures – for example, by starting work earlier, shifting labor to cooler periods, or modifying task intensity.

### Work capacity and physiological response

Rising temperatures impose substantial physiological strain on outdoor workers, with subsistence farmers among the most affected. Evidence from Bawku East, Northern Ghana, shows that during periods of high heat, farmers often work up to nine hours per day with insufficient rest, resulting in adverse health and productivity outcomes [51]. Similarly, a systematic review by Levi et al. (2018) underscores the increased risk of Mesoamerican nephropathy, work-related injuries, and vector-borne diseases linked to heat exposure – factors that directly compromise productivity in labor-intensive sectors such as agriculture [52]. These findings highlight the urgent need for evidence-based policy measures and workplace protections to mitigate health risks in a warming climate.

Leveraging consumer-grade wearable devices, this study will generate high-resolution physiological and behavioral data to examine how heat exposure influences daily activities and health outcomes among farmers. As ambient temperatures continue to rise, we anticipate a decline in physical labor capacity, particularly during peak heat hours, which may manifest as shortened working periods, altered sleep patterns [53], and increased risks of heat stress, dehydration [54,55], and worsening of pre-existing morbidities [9].

Given the limitations of wearable devices in directly measuring dehydration and mental health outcomes, these conditions will be assessed through a combination of self-reported symptoms, behavioral observations, and complementary environmental indicators. The integration of wearable-derived metrics with contextual variables will be critical to developing a holistic understanding of how heat stress affects agricultural workers and to informing the design of targeted adaptation strategies that enhance health and productivity under climate stress.

### Farming behaviour

As climate change continues to reshape agricultural practices, this study will equip each farmer with a Renkforce GP-102 GPS logger to track daily movements and work activities in georeferenced field locations throughout the workday. The resulting spatial-temporal data will provide insight into farming behavior patterns, including harvesting schedules, time allocation for fieldwork, and adaptive responses to heat exposure.

We anticipate that behavioral adaptations to thermal stress may manifest as earlier work start times, longer rest periods, or even shifts toward evening or nocturnal labor to avoid excessive heat. While these adjustments may offer short-term relief, they may also introduce new health risks, such as increased cumulative heat strain, dehydration, and disrupted recovery and sleep patterns. Understanding these evolving work routines is essential for developing evidence-based health guidelines and targeted interventions that promote safe and sustainable labor practices under rising thermal stress.

Beyond individual coping strategies, study findings will contribute to broader climate resilience efforts by informing agricultural adaptation strategies – such as adjusted cropping calendars, crop diversification, and enhanced water management techniques – that can help sustain food production in increasingly variable climate conditions. Furthermore, educational programs to equip farmers with climate-adaptive knowledge and tools will be critical to maintaining productivity and reducing heat-related health burdens.

Supporting this concern, Yengoh and Ardö (2020) projected rising WBGT levels in East Africa using general circulation models, estimating that by 2050 and 2100, farmers in Kenya and Tanzania may need to rest for up to 75% of each hour worked to prevent heat-related illness [56]. While these projections focus on East Africa, similar patterns of heat stress are expected in West Africa, underscoring the need for regionally tailored adaptation and mitigation strategies to preserve labor capacity, agricultural productivity, and food security in the face of accelerating climate change.

### Harvest outcome

Agricultural yield is strongly influenced by weather-related factors, with rising temperatures and climate variability posing significant threats to food security. Kotir (2011) demonstrated that warming temperatures could substantially reduce major cereal yields in sub-Saharan Africa (SSA) [57]. Serdeczny et al. (2017) projected that SSA will experience increased aridity, extreme heat, and altered rainfall patterns due to climate change, which could exacerbate undernutrition rates [28]. Given that a large segment of the population in Burkina Faso and Kenya depends on rainfed agriculture, these climatic shifts place subsistence farming communities at high risk [28]. As agricultural systems become less predictable, subsistence farmers may struggle to maintain stable harvests, potentially leading to increased rural-to-urban migration and worsening nutritional outcomes, particularly for children [28]. Sultan and Gaetani (2016) emphasized that West Africa’s reliance on rainfed agriculture, coupled with limited adaptive capacity, makes it particularly vulnerable to climate change [21]. Both Burkina Faso and Kenya are experiencing climatic shifts, characterized by rising temperatures, changes in monsoonal rainfall patterns, and more frequent climate extremes [21]. There is compelling evidence that agricultural yields in West Africa are already declining due to increasing temperatures, with variable rainfall and elevated CO2 levels further affecting production [21]. These reductions in harvest yields directly impact subsistence farmers, with cascading effects on nutritional status and food security [21]. Food security and climate resilience are intricately linked to agricultural practices and adaptation strategies. Douxchamps et al. (2015) highlighted that crop diversification, conservation agriculture, agroforestry, livestock integration, improved crop varieties, and fertilizers can enhance farm productivity and food security in West Africa [5]. The study categorized households into four types – subsistence, diversified, extensive, and intensified farming systems – each with different levels of food security and vulnerability [5].

These findings underscore the importance of targeted interventions to support climate-resilient agricultural practices and ensure stable food production under shifting environmental conditions. In this context, the use of remote sensing for crop yield modeling, as proposed in this study, offers significant advantages for forecasting agricultural production. Unlike ground-based monitoring, which is resource-intensive and financially unfeasible at scale, remote sensing enables continuous observation of crop health and growth patterns. By integrating satellite-derived vegetation indices and climatic variables, this approach can enhance early warning systems, support climate adaptation strategies, and inform policy decisions aimed at mitigating agricultural losses in vulnerable farming communities.

### Child undernutrition

Heat-induced declines in agricultural productivity can have severe consequences for food security and child nutrition, with children under five being particularly vulnerable. Thiede and Strube (2020) analyzed the relationship between climate anomalies and child undernutrition across 18 sub-Saharan African countries, finding that higher temperatures and reduced rainfall correlated with lower child weight and an increased risk of wasting [58]. These findings highlight the climate sensitivity of child nutrition, emphasizing the need for targeted interventions to mitigate undernutrition in the face of increasing climatic variability [58]. Van der Merwe et al. (2022) systematically examined climate change’s impact on undernutrition, demonstrating that shifts in climate patterns exacerbate child malnutrition risks [36]. Similarly, Phalkey et al. (2015) focused on climate-induced childhood malnutrition, particularly highlighting the increased prevalence of stunting among subsistence farming households in economically disadvantaged regions [34]. These studies reinforce the urgent need for proactive strategies to safeguard child nutrition amid changing agricultural conditions.

To assess how shifts in farming patterns influence food security and nutritional outcomes, this study will equip each farmer with a GPS logger (Renkforce GP-102) to document daily movements and work activities throughout an entire workday. However, wearable data alone cannot directly establish causal links between climate change and child undernutrition. Therefore, this study will integrate GPS-tracked agricultural labor patterns, climate exposure data, and child anthropometric measurements to develop a comprehensive dataset that accounts for multiple influencing factors [34].

The H2H study will evaluate anthropometric indicators, such as height and weight measurements, to generate insights into the nutritional status of children in farming households. Expected findings will explore the linkages between agricultural outcomes and child nutrition, shedding light on how indirect climatic effects, such as reduced harvest yields and food insecurity, contribute to undernutrition risks. To strengthen the interpretation of these relationships, the study will also consider household socioeconomic status, dietary patterns, and maternal health, ensuring a multifaceted analysis of child nutritional vulnerability in the context of climate change.

### Limitations

The climatic and socio-economic context of the study sites – Nouna in Burkina Faso and Kisumu in Kenya – may limit the generalizability of findings to other regions with differing environmental conditions, agricultural systems, or livelihood structures. Additionally, several farming adaptations to heat exposure, such as modifying work hours, are likely long-standing practices. As such, careful interpretation is required to distinguish novel behavioral responses from established coping mechanisms in the face of climatic stress.

The study’s reliance on technological tools, including wearable devices and GPS loggers, introduces potential measurement errors due to technical malfunctions, data synchronization challenges, and variability in participant compliance. While wearables provide detailed physiological data, they are unable to directly measure dehydration or mental health impacts, necessitating reliance on self-reported symptoms, behavioral observations, and contextual data to infer these outcomes.

Our sampling criteria, which include only households with both a resident farmer and children under five, may introduce selection bias, potentially limiting insights from farming households without young children. Furthermore, data collection during the rainy season only may restrict the study’s ability to capture seasonal variations in heat exposure and agricultural productivity, which are critical for understanding year-round stress dynamics.

As the study integrates diverse data streams – including remote sensing, physiological monitoring, and household surveys – there is an inherent risk of data inconsistencies or integration challenges. Moreover, external factors such as market volatility, pest outbreaks, and land-use changes, which significantly affect agricultural outputs, may not be fully captured in the analytical models. Similarly, the focus on anthropometric indicators to assess child undernutrition may overlook important dimensions such as micronutrient deficiencies and dietary quality, which also shape child health outcomes.

While the LASSO regression model offers a robust framework for estimating crop yields, its predictive performance is dependent on specific model assumptions and may not fully reflect the complex interactions between climate variability, soil fertility, and farm management practices. These limitations highlight the need to interpret findings within the broader ecological and socio-economic context that influences agricultural productivity, labor capacity, and health outcomes.

### Recommendations for future research

Future research should consider incorporating livestock health and productivity, given their critical role in food security and rural livelihoods [59]. Integrated crop-livestock systems have been shown to be productive, sustainable, and climate-resilient, offering an alternative to intensive monoculture farming. Such systems can enhance crop yields while improving food security metrics, such as food consumption scores and dietary diversity for rural households [59]. This could represent an additional health impact pathway, linking livestock productivity, economic resilience, and nutritional outcomes. Livestock also serve as a financial safety net during crop failures, acting as a living savings account that supports household stability in times of economic hardship [60].

Adapting the H2H methods to other local contexts is essential for developing tailored climate adaptation strategies that consider regional variations in crop types, farming practices, and climate conditions. For example, GPS tracking of farmers’ movements and wearable monitoring of physiological responses could be replicated across different agricultural settings to assess heat stress impacts on work capacity and health. Extending the H2H methodology to South Asia, where agriculture is also a primary economic driver and food security determinant, could offer valuable insights. Given the region’s distinct climatic patterns, including monsoonal weather and the prevalence of rice paddies, models used in remote sensing-based crop yield forecasting and work capacity assessments would need to be calibrated for local environmental conditions. Such adaptations could inform policy recommendations on irrigation practices, heat-tolerant crop varieties, and the timing of agricultural activities to minimize heat stress impacts on productivity.

Additionally, future research should investigate the prevalence and local distribution of non-communicable diseases (NCDs), such as chronic obstructive pulmonary disease (COPD) and asthma, as these conditions can significantly impact physical work capacity. Understanding the interaction between pre-existing health conditions, heat exposure, and labor capacity will be crucial for designing comprehensive adaptation strategies that address both environmental and health-related vulnerabilities. Given that recommendations for future research should be informed by study findings, subsequent studies should build upon empirical evidence from the H2H study to refine intervention strategies and policy recommendations in a data-driven manner.

## Conclusion

This paper outlines the methodological framework for the Heat to Harvest (H2H) study, detailing its interdisciplinary approach to investigating the health and agricultural impacts of climate change on farming communities in sub-Saharan Africa. As a feasibility study, this phase will generate critical insights to inform the design, implementation, and refinement of the main H2H study, enhancing our understanding of the causal pathways through which climate variability affects agricultural livelihoods, physiological health, and child nutrition.

The study will examine the interconnected effects of heat and humidity on farmers’ physiological responses, work capacity, agricultural productivity, and nutritional outcomes, identifying key drivers of vulnerability and adaptation. Given that farmers have long employed adaptive practices such as shifting work hours, the study will assess whether current behavioral changes represent novel strategies or continuations of long-standing responses to environmental stressors.

The overarching aim of the H2H study is to generate actionable evidence on the health and food security implications of heat stress, with a view toward informing climate-resilient agricultural strategies, targeted health interventions, and adaptive policy frameworks. Through the integration of wearable technology, GPS tracking, remote sensing, and household health assessments, the study will provide a robust evidence base to support sustainable farming practices and climate adaptation planning.

Findings from the full H2H study will contribute to strengthening the resilience of agricultural communities, improving household food security, and guiding policy efforts to protect health and livelihoods in the context of a warming and increasingly unpredictable climate.
